# Neuronal Lipid Metabolism: Multiple Pathways Driving Functional Outcomes in Health and Disease

**DOI:** 10.3389/fnmol.2018.00010

**Published:** 2018-01-23

**Authors:** Timothy J. Tracey, Frederik J. Steyn, Ernst J. Wolvetang, Shyuan T. Ngo

**Affiliations:** ^1^Australian Institute for Bioengineering and Nanotechnology, The University of Queensland, Brisbane, QLD, Australia; ^2^Centre for Clinical Research, The University of Queensland, Brisbane, QLD, Australia; ^3^Queensland Brain Institute, The University of Queensland, Brisbane, QLD, Australia

**Keywords:** lipid metabolism, neuronal metabolism, amyotrophic lateral sclerosis, mitochondria, glycosphingolipid

## Abstract

Lipids are a fundamental class of organic molecules implicated in a wide range of biological processes related to their structural diversity, and based on this can be broadly classified into five categories; fatty acids, triacylglycerols (TAGs), phospholipids, sterol lipids and sphingolipids. Different lipid classes play major roles in neuronal cell populations; they can be used as energy substrates, act as building blocks for cellular structural machinery, serve as bioactive molecules, or a combination of each. In amyotrophic lateral sclerosis (ALS), dysfunctions in lipid metabolism and function have been identified as potential drivers of pathogenesis. In particular, aberrant lipid metabolism is proposed to underlie denervation of neuromuscular junctions, mitochondrial dysfunction, excitotoxicity, impaired neuronal transport, cytoskeletal defects, inflammation and reduced neurotransmitter release. Here we review current knowledge of the roles of lipid metabolism and function in the CNS and discuss how modulating these pathways may offer novel therapeutic options for treating ALS.

## Introduction

Lipids are fundamental organic molecules that are utilized by the human body for a number of essential cellular processes. Broadly speaking, lipids can be classified into five major subcategories. These include fatty acids, triglycerides, phospholipids, sterol lipids and sphingolipids. Significant diversity in lipid structure exists, with between 1000 and 2000 different lipid species thought to exist in mammalian biological systems. The importance of lipids to biological systems is highlighted by the fact that 5% of all human genes are devoted to lipid synthesis (van Meer et al., 2008). The brain makes great use of all five classes of lipids, and contains the second highest concentration of lipids in the human body (Hamilton et al., 2007). In this review, we discuss their individual structure, synthesis, and transport in the context of brain function. We then discuss their roles in amyotrophic lateral sclerosis (ALS) pathogenesis and discuss how modulating lipid function may offer novel therapeutic options.

## Lipid Synthesis, Structure and Transport

**Figure 1:**
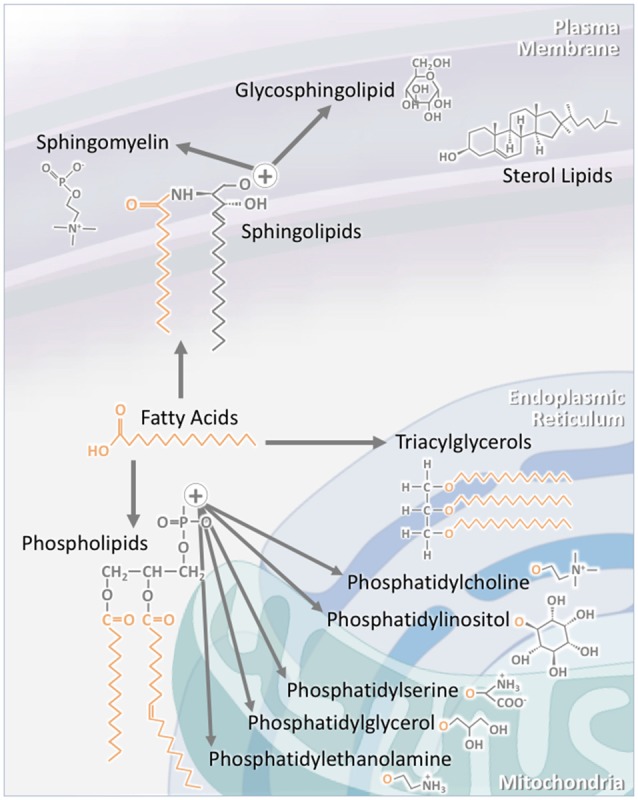
The structure of individual lipid classes affects their intracellular localization. Triacylglycerols (TAGs) consist of three fatty acid chains attached to a glycerol backbone. As TAG synthesis occurs in the endoplasmic reticulum (ER), a large proportion of TAGs are found within this compartment. A significant proportion is also stored in specialized intracellular organelles known as lipid droplets. The subclasses of phospholipids are synthesized through a number of pathways. Phosphatidylcholine and phosphatidylinositol are largely localized to the ER, while phosphatidylserine, phosphatidylglycerol and phosphatidylethanolamine are mainly to localized to the mitochondria and its associated membranes. Sterol lipids, such as cholesterol, are highly concentrated at the plasma membrane. Sphingolipids are also largely localized to the plasma membrane. This is particularly the case for sphingomyelin and glycosphingolipids. As major components of lipid rafts, sphingolipid concentration at the plasma membrane is kept high.

In this section of the review, we provide an overview of the synthesis, structure, and transport of fatty acids, triacylglycerols (TAGs), phospholipids, sterol lipids and sphingolipids. The intracellular localization of the individual lipid classes is summarized in Figure 1.

### Fatty Acids

#### Fatty Acid Structure

As the essential monomeric component of all lipid types, fatty acids are an essential class of lipids. All fatty acids consist of a carbon chain that terminates in a carboxylic acid functional group, and they are categorized into different subclasses based on the length of the carbon chain. Fatty acids with 2–4 carbons are classified as short-chain fatty acids, medium-chain fatty acids have 6–12 carbons, long-chain fatty acids have 14–18 carbons, and very long-chain fatty acids have 18+ carbons. The length of the fatty acid introduces significant variation in function and subcellular localization (Agostoni and Bruzzese, 1992).

In fatty acids, the carbon chain is classified as either saturated or unsaturated. A saturated fatty acid is defined by a carbon chain in which all of the carbons are “saturated” with hydrogen atoms. As such, only single bonds exist between the carbons. An unsaturated fatty acid, however, contains a carbon chain in which double bonds have been introduced. Depending on the number of double bonds, the fatty acid can be classified as mono- or polyunsaturated. Polyunsaturated fatty acids (PUFAs) are of particular importance in the brain, where they serve as essential molecules for signaling and membrane structure (Bazinet and Layé, 2014). Another variation, although rare in humans, is the branching, or functionalization, of the fatty acid chain. For example, a saturated fatty acid has one or more methyl groups added to the carbon chain, significantly altering their physical properties, and, consequentially, ordering in cellular membranes (Ran-Ressler et al., 2008).

The most common modification to fatty acids is microsomal fatty acid elongation, which predominantly takes place on the endoplasmic reticulum (ER). Malonyl CoA acts as the major substrate, and undergoes repeated cycles of condensation, reduction, dehydration and reduction reactions (Bressler and Wakil, 1962). Unlike the cytosolic process, which uses one enzyme complex only, microsomal chain elongation is coordinated by the action of four unique enzymes. The process is regulated primarily at the condensation step by the 3-keto acyl-CoA synthase enzyme (Moon and Horton, 2003). Seven forms of the enzyme have been characterized in humans, and each utilizes different input fatty acids, resulting in varying degrees of chain elongation (Leonard et al., 2004; Jakobsson et al., 2006). Chain elongation can also take place in the mitochondria, but this is far less common. The fatty acid oxidation pathway that typically takes place in the mitochondria is reversed. Since this process is energetically unfavorable to reverse, the acyl-CoA dehydrogenase step is substituted for enoyl-CoA reductase. Such elongation also occurs in the peroxisomes, in a similar fashion to the mitochondria (Alexson and Cannon, 1984; Wong et al., 2004; Demarquoy and Le Borgne, 2015).

Fatty acids can also be modified through desaturation, a process that introduces double bonds into the fatty acid chain. Depending on the desaturase enzyme used, the position and number of double bonds differs. The most common desaturases are ∆9, ∆5 and ∆6, along with fatty acid desaturase 3 (FADS3; Nakamura and Nara, 2004). Desaturation is essential for diversifying fatty acid structure and function.

#### Fatty Acid Synthesis

**Figure 2:**
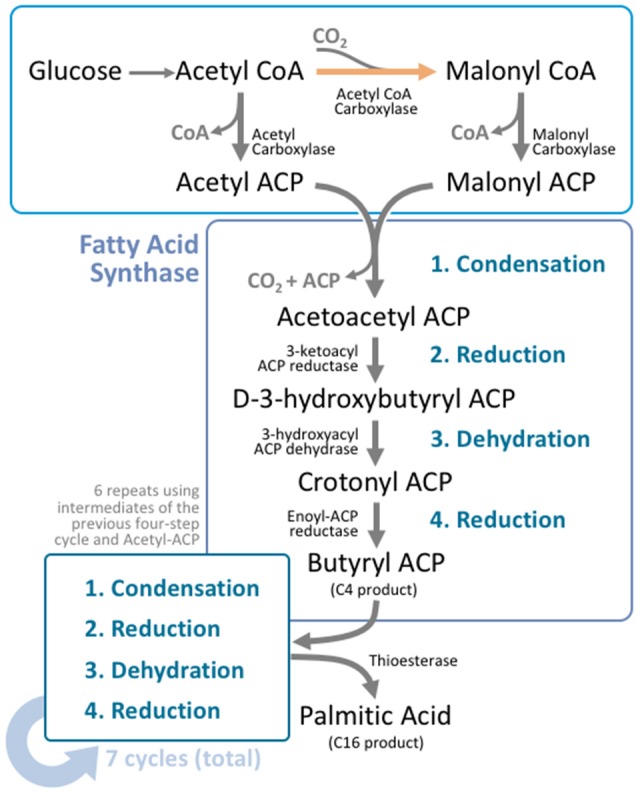
Fatty acid synthesis is a cyclical process that utilizes a unique multifunctional enzyme complex. Glucose is metabolized to acetyl CoA through the standard glucose metabolic pathway. Acetyl CoA is then carboxylated by acetyl CoA carboxylase (ACC) to form malonyl CoA. This carboxylation step is the rate limiting process in fatty acid synthesis. Malonyl CoA and unreacted acetyl CoA undergo transacylation to form malonyl ACP and acetyl ACP respectively. These intermediates enter fatty acid synthase (FAS), where four reactions take place; condensation, reduction, dehydration, and a second reduction. The four reactions add two carbons to the original carbon chain—in this case malonyl ACP. In humans, this cycle is typically repeated six more times, using the product from the end of the cycle as the input molecules for the next cycle, producing palmitoyl ACP; a 16 carbon product. A thioesterase enzyme then terminates the carbon chain at the thioester bond, forming palmitic acid.

Fatty acids are synthesized in the cytosol of lipogenic tissues. While the brain can synthesize the majority of required saturated and monosaturated fatty acids, it severely lacks the ability to synthesize PUFAs (Moore, 2001). Fatty acid synthesis takes place over seven repetitions of a four-reaction cycle (Figure 2). Acetyl CoA, which is provided through the metabolism of glucose, is first carboxylated to malonyl CoA via acetyl CoA carboxylase (ACC; Wakil et al., 1983). In humans, there are two major forms of ACC: ACC1 and ACC2 (Abu-Elheiga et al., 1995, 1997; Ha et al., 1996). ACC1 is the minor form expressed in the cytosol of human tissues, while ACC2 is the major form, and is expressed on the mitochondrial membrane (Castle et al., 2009). Expression is observed most highly in lipogenic tissues, such as adipose tissue and the liver, along with oxidative tissues, such as skeletal muscle and the heart (Kreuz et al., 2009). ACC2 is also highly expressed in the brain and spinal cord (Castle et al., 2009). The carboxylation of acetyl CoA to malonyl CoA is irreversible and the rate-limiting step in fatty acid synthesis (Ha and Kim, 1994).

After this carboxylation process, malonyl CoA acts as a carbon donor in the fatty acid synthesis process (Figure 2). The unreacted acetyl CoA and malonyl CoA intermediates undergo transacylation to form acetyl ACP and malonyl ACP respectively. These serve as the starting point from which the four-reaction cycle repeats, and is facilitated by fatty acid synthase (FAS). FAS is a homodimer found within the cytoplasm that contains three domains and seven catalytic sites (Chirala et al., 2001). The first domain acts as the substrate entry and condensation unit, the second domain acts as the reduction unit, and the third domain acts as the fatty acid exit domain. As a result, the entirety of fatty acid synthesis takes place within a single enzyme (Wakil, 1989; Smith, 1994).

The first reaction process is condensation. Acetyl ACP and malonyl ACP react to form acetoacetyl ACP, which is catalyzed by acyl-malonyl ACP condensing enzyme. Acetoacetyl ACP then undergoes reduction, to form D-3-hydroxybutyryl ACP, which is then dehydrated to form crotonyl ACP. Finally, crotonyl ACP is reduced to butyryl ACP, which marks the end of the first of seven elongation steps. As such, the first round of elongation ends with a 4-carbon chain product. Subsequent elongation cycles extend this carbon chain by two carbons each time, until a 16 carbon-acyl ACP (palmitoyl ACP) is formed, after seven repetitions. At this stage, a thioesterase enzyme catalyzes the formation of palmitic acid by terminating the carbon chain at the thioester bond. As a result, fatty acid synthesis is terminated with the formation of palmitic acid (Figure 2).

Rather than relying solely on *de novo* synthesis and modification, the majority of fatty acids are provided from the diet. Short and medium-chain fatty acids are of particular importance as *de novo* synthesis is focused on the production of long and very long-chain fatty acids. Dietary fatty acids are mobilized directly into the bloodstream as triglycerides, and stored in adipocytes until required by the body. There are two fatty acids for which the body cannot synthesize sufficient quantities, and must rely on dietary sources. These are linoleic acid and α-linolenic acid, which are referred to as essential fatty acids (Ellis and Isbell, 1926). These dietary fatty acids are also susceptible to modification, further diversifying the fatty acid pool. The major products of linoleic acid and α-linolenic acid are arachidonic acid and docosahexaenoic acid—the major brain PUFAs, making a diet rich in essential fatty acids important for healthy brain function (Bazinet and Layé, 2014).

#### Fatty Acid Transport

Due to the functional importance of PUFAs in the brain, a significant portion of fatty acids must be imported from the blood. In such cases, fatty acids are metabolized from sources in adipose tissue or the bloodstream by lipoprotein lipase (LPL; Goldberg et al., 2009). These “free fatty acids” then bind albumin in the blood as a carrier protein, and are transported throughout the circulatory system (Korn, 1955a,b). Upon reaching the CNS, the fatty acids must pass the blood brain barrier.

Fatty acids are postulated to pass through the blood brain barrier by two mechanisms. In the passive diffusion model, fatty acids are hypothesized to dissociate from their albumin carriers and bind to the luminal membrane of the endothelial cell. Once bound, the fatty acids diffuse across the membrane in a non-ATP-dependent manner and enter the cytosol. This process is repeated for the transluminal membrane, allowing the fatty acids access to the brain extracellular space. From this point, they cross the plasma membrane of the neural cells, and reach their target. This is referred to as the flip-flop method (Simard et al., 2008). It is argued that this diffusion process is dependent on the lipophilicity and size of the fatty acid (Kampf et al., 2006). It has been shown that short and medium-chain fatty acids easily cross the blood brain barrier due to their high permeability coefficients, while long-chain fatty acids are less permeable, and need to be in their non-ionized form for faster movement (Kamp and Hamilton, 1992). Such differential diffusion speeds have served as the major criticism of the diffusion model, where the significantly slower diffusion of the long-chain fatty acids may not be sufficient to supply the metabolic needs of the CNS. Thus, faster ATP-dependent transporter protein-mediated mechanisms have been postulated. Involving four classes of transport proteins: Fatty acid transport protein (FATP), fatty acid translocase, Fatty acid binding proteins (FABPs) and caveolae, these mechanisms can theoretically support the high fatty acid metabolic rate of the brain (Mitchell and Hatch, 2011).

FATP consists of six tissue specific isoforms. FATP-1 and FATP-4 are the major isoforms found in the brain, and act on the luminal membrane of the endothelial cells (Mitchell et al., 2011). FATP-1 possesses specificity towards long chain fatty acids (Watkins et al., 1998; Mitchell et al., 2011). ATP-dependent transport of fatty acids has also been linked to long-chain acyl-CoA synthetase, marking it as an essential complex in multiple aspects of fatty acid metabolism.

Fatty acid translocase/CD36 shows specificity for the long and very long-chain fatty acids (Pepino et al., 2014). High levels of expression are also observed in the brain, including on the luminal membrane of brain endothelial cells (Husemann et al., 2002). While the mechanism by which CD36 transports fatty acids is not fully understood, studies suggest that CD36 promotes uptake via modifications to intracellular metabolism (i.e., esterification), rather than directly increasing the rate of fatty acid transport across the plasma membrane (Xu et al., 2013).

FABPs are made up of two subfamilies. Membrane-associated FABPs are associated with the extracellular surface of the plasma membrane, and bind all fatty acids with high affinity. The mechanism of membrane-associated FABP-mediated transport is not wholly understood, but it is hypothesized that they are not directly involved in transport, but rather act as an intermediator between the free fatty acids and FATPs. Cytosolic FABPs are more deeply understood. Multiple subtypes of cytosolic FABPs show tissue specific expression (Glatz et al., 1997). Membrane transport of cytosolic FABP is hypothesized to occur by one of two mechanisms. The first is by aqueous phase diffusion through the membrane, where cytosolic FABP-fatty acid complexes passively diffuse through the plasma membrane, and the other is through collision transfer, where the FABP-fatty acid complex makes contact with the membrane to transfer the fatty acid (Storch and Thumser, 2000).

Caveolae are intracellular invaginations of the plasma membrane, for which three isoforms exist. Caveolin-1 has been shown to have a major role in the transport of long-chain fatty acids (Pohl et al., 2002, 2005), and it is proposed that the caveolar domain is capable of budding off to form intracellular vesicles, which then carries the fatty acids to subcellular organelles for further processing (Stremmel et al., 2001). Fission of the vesicles with the plasma membrane transfers the lipid cargo from one membrane to another. Caveolin is also postulated to indirectly modulate fatty acid uptake by controling the localization of CD36 (Lobo et al., 2001; Ring et al., 2006).

Therefore, fatty acids enter the brain through a number of different pathways; either via passive diffusion, or a host of transport-protein mediated pathways. As a result, the brain has access to a range of fatty acids for metabolic purposes.

### Triacylglycerol

Compared to the other lipid classes, TAGs play a small role in neuronal lipid metabolism. Nevertheless, they do act as the storage form of lipid precursors.

#### Triacylglycerol Structure

TAGs are composed of a glycerol backbone with three fatty acid chains. Variation in TAG structure arises from the fatty acid chains, which can vary in length, functionalization and degree of saturation. The position at which these fatty acids are added to the glycerol backbone affects the physical and physiological properties of the TAG (Karupaiah and Sundram, 2007).

#### Triacylglycerol Synthesis

The synthesis of TAG primarily occurs in adipose tissue and the liver, but is also observed in skeletal muscle, kidney, lung, heart and the brain. TAG synthesis can occur via the glycerol-3-phosphate, or the monoacylglycerol pathway in both the ER and the mitochondria (Weiss et al., 1960; Lehner and Kuksis, 1996; Dircks and Sul, 1999). In the glycerol-3-phosphate pathway, synthesis begins with glucose, which is converted into glycerol-3-phosphate by a multi-step metabolic reaction. Glycerol-3-phosphate, the rate-limiting step of TAG synthesis, is converted to lysophosphatidic acid by glycerol-3-phosphate acyltransferase (GPAT). Lysophosphatidic acid is then converted into phosphatidic acid by 1-acylglycerol-3-phosphate acyltransferase (AGPAT), phosphatidic acid into 1,2-diacylglycerol by phosphatidic acid phosphatase (PAP), and finally 1,2-diacylglycerol into TAG by diacylglycerol acyltransferase (DGAT). The monoacylglycerol pathway is similar, but rather than initiating with glucose, this pathway begins with monoacylglycerol, which is converted into 1,2-diacylgycerol by monoacylglycerol acyltransferase (MGAT). The pathway then continues as for glycerol-3-phosphate. After synthesis, TAGs are packaged into lipid droplets (Coleman and Lee, 2004).

TAG breakdown; a process also known as lipolysis, is essential for the presentation of fatty acids to multiple tissue types. Lipolysis principally occurs in the adipose tissue and is facilitated by a set of enzymes known as lipases (Ahmadian et al., 2007). Lipolysis begins with the conversion of TAG into diacylglycerol by adipose triglyceride lipase (ATGL), releasing one fatty acid molecule (Zimmermann et al., 2004). This reaction can also be catalyzed to some extent by hormone-sensitive lipase (HSL). Diacylglycerol is then further catabolized to monoacylglycerol by HSL, releasing a second fatty acid molecule (Haemmerle et al., 2002). Finally, monoacylglycerol is broken down to glycerol and the final fatty acid (Fredrikson et al., 1986). Lipolysis can also occur in the bloodstream, as is required for the presentation of fatty acids to the blood brain barrier. In such cases, LPL catalyzes the reaction (Goldberg, 1996).

#### Triacylglycerol Transport

While TAG synthesis can take place in the brain, liver, heart, skeletal muscle and kidney, they are also transported to these regions through the bloodstream. In order to be transported, they must be packaged into lipoproteins. Lipoproteins are composed of a hydrophobic core of TAGs, cholesterol esters and fat soluble vitamins, which are enveloped by a layer of phospholipids, free cholesterol, and specialized apolipoproteins. There are multiple subtypes of lipoprotein, and they are categorized as high-density (HDL), low-density (LDL), intermediate-density (IDL) and very low-density lipoproteins (VLDL) based on their density. Another type of lipoprotein are the chylomicrons, and the size of these particles is controlled primarily by the number of TAG molecules that are incorporated (Jackson et al., 1976).

The majority of TAGs are transported by the chylomicrons and VLDLs. Chylomicrons transport TAGs from dietary sources, while VLDLs deliver TAGs from endogenous sources (Dole and Hamlin, 1962; Zilversmit, 1967; Jackson et al., 1976). A number of other apolipoproteins are found on the lipoproteins surface, and act as ligands and modulators of receptor and enzyme activity. Once lipoproteins reach their target tissue, they are catabolized by LPL, releasing free fatty acids for cellular uptake, or can bind to lipoprotein receptors on the cell surface, leading to immediate incorporation (Goldberg, 1996).

### Phospholipids

Phospholipids are a diverse class of lipids, with a wide range of roles in the human body. In particular, they hold great functional importance in the brain. Phospholipids can be generally categorized into two groups; the glycerophospholipids, and the phosphosphingolipids. Glycerophospholipids can be further categorized into phosphatidylcholine, phosphatidylethanolamine, phosphatidylserine, phosphatidylinositol, phosphatidylglycerol and cardiolipin (Li et al., 2015). Phosphosphingolipids will be considered in the context of sphingolipids later in this review.

#### Phospholipid Structure

Phospholipids are composed of a glycerol backbone, with a hydrophobic fatty acid tail, a hydrophilic head group, and a phosphate group. The type of head group identifies the phospholipid, with choline, ethanolamine, serine, inositol, or glycerol groups capable of addition (Li et al., 2015). This hydrophobic/hydrophilic polarity defines the phospholipids as amphipathic.

#### Phospholipid Synthesis

**Figure 3:**
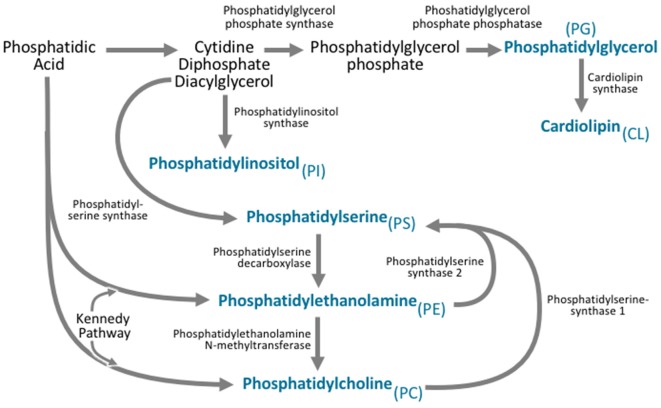
All phospholipid subclasses are derived from phosphatidic acid via a variety of synthetic pathways. Phosphatidylcholine is synthesized via two pathways. The major pathway is the Kennedy pathway and involves the addition of CDP-choline to the phosphatidic acid derivative diacylglycerol. Phosphatidylcholine can also be synthesized through the action of phosphatidylethanolamine N-methyltransferase (PEMT), which converts phosphatidylethanolamine to phosphatidylcholine. Phosphatidylethanolamine is also synthesized via the Kennedy pathway, where the multifunctional enzymes catalyze the addition of CDP-ethanolamine to diacylglycerol. Phosphatidylethanolamine can also be synthesized by phosphatidylserine decarboxylase. Phosphatidylserine is synthesized from CDP-diacylglycerol, phosphatidylcholine, and phosphatidylethanolamine via phosphatidylserine synthase. Phosphatidylinositol is derived from the CDP-diacylglycerol by phosphatidylinositol synthase. Phosphatidylglycerol is synthesized from CDP-diacylglycerol via a multistep process involving phosphatidylglycerol phosphate synthase, and phosphatidylglycerol phosphate phosphatase. Cardiolipin can then be synthesized from phosphatidylglycerol via cardiolipin synthase.

Synthesis of all classes of phospholipids begins with two common precursors: phosphatidic acid and diacylglycerol (Figure 3). Phospholipid synthesis largely occurs in the ER in all major tissue types (Vance et al., 1977; Coleman and Bell, 1978; Fagone and Jackowski, 2009). In particular, the brain takes part in a substantial amount of phospholipid synthesis (Ross et al., 1997).

Phosphatidylcholines are synthesized through two major pathways (Figure 3). The first is the CDP-Choline/Kennedy pathway, through which the majority of *de novo* synthesis takes place (Kennedy and Weiss, 1956; Weiss et al., 1958). The second is the phosphatidylethanolamine N-methyltransferase (PEMT) pathway, which synthesizes phosphatidylcholines by the successive methylation of the phospholipid phosphatidylethanolamine (Shields et al., 2003; Hörl et al., 2011).

Phosphatidylethanolamine synthesis occurs through the Kennedy pathway (Wright and McMaster, 2002; Figure 3). The enzyme CTP:phosphoethanolamine cytidylyltransferase is the exception to this, and utilizes only phosphatidylethanolamine synthesis substrates (Vermeulen et al., 1993). While this form of synthesis takes place in the ER, phosphatidylethanolamine synthesis can also occur on the inner mitochondrial membrane, through the phosphatidylserine decarboxylase pathway (Dennis and Kennedy, 1972).

Synthesis of phosphatidylserine is a process that relies on phosphatidylcholines and phosphatidylethanolamines (Figure 3). Phosphatidylserine is synthesized when the head group of either phosphatidylcholines or phosphatidylethanolamines is replaced by serine. Phosphatidylserine synthase 1 catalyzes this reaction for phosphatidylcholines, while phosphatidylserine synthase 2 catalyzes this reaction for phosphatidylethanolamines (Kuge et al., 1986). These reactions take place in the mitochondria-associated membranes of the ER (Stone and Vance, 2000).

Phosphatidylinositol synthesis relies on phosphatidylinositol synthase to convert CDP-diacylglycerol—a modified version of diacylglycerol, directly to phosphatidylinositol (Antonsson, 1997; Figure 3). This process occurs at the ER, but can also occur in ER-derived vesicles as well as the plasma membrane (Kim et al., 2011). Phosphatidylinositol is then heavily modified to produce a number of essential signaling molecules, the importance of which will be discussed later.

CDP-diacylglycerol is also essential for the synthesis of phosphatidylglycerol and cardiolipin (Figure 3). On the inner membrane of mitochondria, CDP-diacylglycerol is converted to phosphatidylglycerol phosphate by phosphatidylglycerol phosphate synthase, which is then further dephosphorylated to form phosphatidylglycerol (Kawasaki et al., 1999, 2001; Scherer and Schmitz, 2011). Phosphatidylglycerol can then be further modified by cardiolipin synthase, to form cardiolipin (Schlame et al., 1993).

#### Phospholipid Transport

While phospholipids are synthesized in their target cells at the ER and mitochondria, intracellular transport is required for phospholipid utilization at appropriate membranes. Phospholipid transport is proposed to occur via soluble transport proteins, vesicular transport, and close membrane contact between “acceptor” and “donor” membranes (Vance, 2015).

Localized within the cytosol, three forms of soluble transport proteins have been characterized. The first is the phosphatidylcholine-specific transfer protein (PC-TP). Labeling studies show that PC-TP is essential for the movement of phosphatidylcholines from the ER to phosphatidylcholine-deficient membranes, yet it does not appear to play a major role in phosphatidylcholine-specific transport (Kanno et al., 2007). The second are phosphatidylinositol transfer proteins α and β (PITPα and PITPβ). While their name suggests phosphatidylinositol-specific action, their proteins are also capable of transporting phosphatidylcholine, although at a much lower rate (Helmkamp et al., 1974). Robust characterization of the action of these proteins is still required, and as such, most suggested functionality is only hypothesized based on localization and structure. The final transport protein is the non-specific lipid transfer protein, which transports all classes of lipids (Bloj and Zilversmit, 1977). Unlike the other transport proteins, expression is also observed in the peroxisomes (Keller et al., 1989; Mendis-Handagama et al., 1992). Knockdown of this protein does not markedly affect phospholipid distribution, suggesting that it does not play a major role in intracellular phospholipid transport (Seedorf et al., 1998). Thus, protein-dependent intracellular transport does not appear to be a major contributor to phospholipid redistribution.

Vesicular phospholipid transport is not well characterized. The rationale behind this model is that vesicles that typically carry proteins to cellular membranes contain a bilayer of phospholipid containing molecules. When these vesicles fuse with the membrane, it is expected that the phospholipids will be incorporated into the new membrane (Vance, 2015). Studies of labeled phosphatidylcholines and phosphatidylethanolamines have shown that the uptake of these phospholipids occurs on a different timescale compared to vesicular protein cargo (Vance et al., 1991; Field et al., 1998; Huijbregts et al., 1998). This suggests that if vesicular uptake of phospholipids at the plasma membrane is occurring, it is happening in non-protein associated vesicles. There also appears to be evidence that the transport of phospholipids from the plasma membrane back to the ER and mitochondria does occur, but this requires further validation (Sleight and Pagano, 1984, 1985).

The final proposed mechanism of phospholipid transport involves close membrane association. In this model, close associations between membranes are formed by the action of multiprotein tethering complexes. Indeed, electron microscopy studies have shown such close associations between the ER and mitochondria (Perkins et al., 1997), as well as the ER and plasma membrane (Pichler et al., 2001). While phospholipid transport between the ER and plasma membrane has yet to be observed in this manner, transport between the ER and mitochondria has been well characterized. Membrane tether-associated transport of phosphatidylcholines, phosphatidylethanolamines and phosphatidylserine has been observed in multiple cell types (Wu and Voelker, 2004; Horibata and Sugimoto, 2010; Tasseva et al., 2013).

### Sterol Lipids

Sterol lipids are an essential class of lipids of particular importance to the brain. Sterol lipids are synthesized in many forms, but the major form in all mammals is cholesterol. For this review, all sterol lipids will be considered in the form of cholesterol.

#### Sterol Lipid Structure

Cholesterol is defined by its tetracyclic ring structure. A hydroxyl group is added to one end of this ring structure, which defines that portion as hydrophilic. The combination of the tetracyclic ring and hydroxyl group designates the sterol portion of the molecule (Tabas, 2002). A hydrocarbon chain is attached to the other end of the sterol. By nature, this hydrocarbon chain is hydrophobic, making cholesterol an amphipathic molecule.

#### Sterol Lipid Synthesis

**Figure 4:**
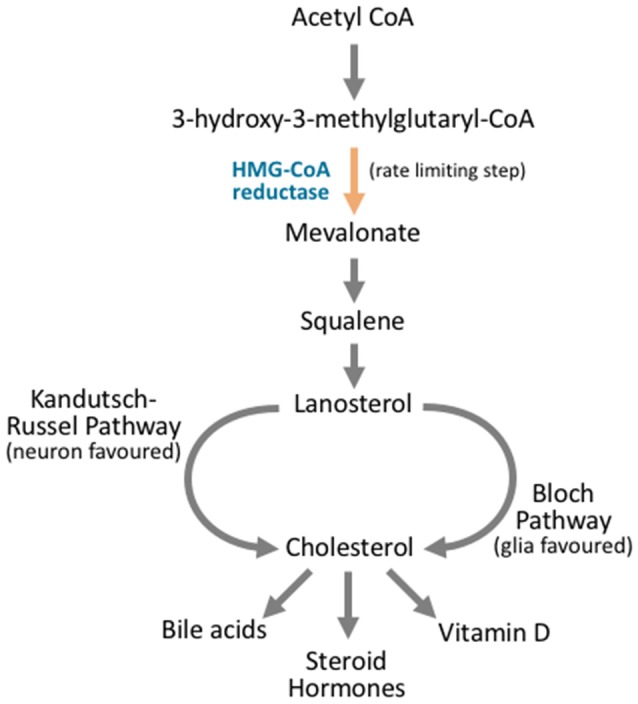
Cholesterol synthesis occurs through diverging pathways for different neuronal cell types. Cholesterol synthesis acts through the mevalonate pathway. Acetyl CoA is converted to 3-hydroxy-3-methylglutaryl CoA (HMG-CoA). HMG CoA is then converted to mevalonate via HMG-CoA reductase, and this is the rate limiting step in cholesterol synthesis. Via a complex series of reactions that involve multiple enzymes and reaction steps, mevalonate is converted to squalene. Squalene is converted to lanosterol in an essential cyclization process. From here, the Kandutsch-Russel pathway, which is favored by neuronal cells, produces cholesterol from lanosterol, while the Bloch pathway synthesizes cholesterol that is favored by glial cells. After cholesterol is synthesized, it can be further metabolized into vitamin D, steroid hormones, or bile acids, or it may be incorporated into cellular membranes.

All nucleated cells are capable of synthesizing cholesterol at the ER. The majority of cholesterol synthesis takes place in the liver, with significant quantities also being produced by the brain, intestines, and adrenal glands (Blom et al., 2011). Cholesterol synthesis acts through the mevalonate pathway, a complex series of reactions that utilize more than 20 steps (Langdon and Bloch, 1953; Bloch, 1965, 1992; Figure 4). Synthesis begins with acetyl CoA, which is converted to 3-hydroxy-3-methylglutaryl-CoA (HMG-CoA), and then mevalonate. The rate-limiting and irreversible step of cholesterol synthesis is this conversion of HMG-CoA to mevalonate by HMG-CoA reductase. As such, it is heavily regulated at both transcriptional and post-translational levels (Ye and DeBose-Boyd, 2011). Mevalonate, via a six enzyme process, is converted to squalene. Lanosterol, the first cyclic intermediate in the synthesis pathway, is produced from squalene. At this stage, the synthesis pathways diverge into the Kandutsch-Russell or Bloch pathways—depending on the nature of the hydrocarbon chain, before converging at the final product of cholesterol (Kandutsch and Russell, 1960; Bloch, 1965). From this point, cholesterol can be used to form bile acids, steroid hormones, or vitamin D (Figure 4).

In the brain, the divergent pathways of cholesterol synthesis take precedence in different neuronal tissues. Neurons contain cholesterol variants that arise from the Kandutsch-Russell pathway, while astrocytes favor Bloch pathway variants. In a similar vein, levels of the intermediate lanosterol are found to be much higher in neurons when compared to astrocytes. Cholesterol levels are also much higher in glial cells (Nieweg et al., 2009). Together these findings have led to suggestions that neurons have lower capacity for cholesterol synthesis (Zhang and Liu, 2015).

#### Sterol Lipid Transport

Cholesterol transport occurs between and within cells. Dietary sources of cholesterol are believed to account for as much as 70% of total body cholesterol. As such, transport is an essential mechanism for appropriate cholesterol distribution. Dietary cholesterol is transported from the gut to the liver, and then distributed throughout the body. For this to occur, intestinal enterocytes package cholesterol into chylomicrons, and liver hepatocytes package cholesterol into VLDLs (Ikonen, 2008). Once in circulation, VLDLs are modified into LDLs, which deliver cholesterol to the peripheral tissues. If cholesterol reaches excess in the periphery, transport mechanisms are required to rebalance the distribution. In such cases, cholesterol is packaged into HDLs, which return it to the liver. In the CNS, lipoproteins are also used to transport cholesterol between multiple cell types (Pitas et al., 1987; Orth and Bellosta, 2012).

While the ER is the major site of cholesterol synthesis, the concentration of cholesterol at the ER is typically low due to the rapid intracellular transport of cholesterol to appropriate membranes (Blom et al., 2011). A significant portion of cholesterol is transported to the plasma membrane via non-vesicular mechanisms. This is proposed to occur via the action of cytosolic carrier proteins, such as sterol carrier protein-2 (SCP-2; Puglielli et al., 1995; Gallegos et al., 2001), the oxysterol-binding protein-related proteins (ORPs; Zhao and Ridgway, 2017), and members of the steroidogenic acute regulatory protein-related lipid transfer (START) family (Gatta et al., 2015). Members of the START family have also been shown to be essential for the transport of cholesterol into the mitochondria (Clark et al., 1994). A small portion of cholesterol is trafficked through the standard biosynthetic Golgi complex secretory pathway, where it is presented to the plasma membrane. Membrane trafficking via intracellular LDL receptor-mediated uptake brings a portion of cholesterol into the endosomal system, where it is recycled to the plasma membrane and mitochondria (Sugii et al., 2003; Wojtanik and Liscum, 2003). A final portion of free cholesterol is esterified to form fatty acid sterol esters, which are packaged into lipid droplets for storage (Robenek et al., 2006; Ploegh, 2007). As a result, intracellular cholesterol transport is in a constant state of flux, with high levels of turnover at the ER.

### Sphingolipids

#### Sphingolipid Structure

Although various forms of sphingolipids exist, all are characterized by the inclusion of a sphingosine backbone. Sphingosine does not apply to a single structure, but is a broad term encompassing various modifications of a long chain base. Long chain bases are either 2-amino-1,3dihydroxyalkanes/2-amino-1,3dihydroxyalkenes, with an alkyl chain between 14 and 22 carbons, and up to 2 double bonds (Chester, 1998). Significant branching and hydroxyl group additions can also occur.

Depending on the class of sphingolipid, a number of different groups can be added to the sphingosine backbone. The simplest sphingolipids are the ceramides, which consist of a sphingosine backbone linked to a fatty acid chain (Pinto et al., 2011). There is significant variation in the attached fatty acid, and mammalian sphingolipids typically contain saturated fatty acid chains of between 14 and 32 carbons (Merrill et al., 2005). Due to the inclusion of this fatty acid chain, sphingolipids are amphipathic.

Other classes of sphingolipids are introduced through the addition of various head groups to ceramide. Sphingomyelins result from the addition of phosphocholine to ceramide (Huitema et al., 2004). The addition of phosphocholine defines these lipids as phospholipids, and they are therefore also referred to as phosphosphingolipids. Glycosphingolipids, also referred to as cerebrosides, arise when one or more sugar residues are added to ceramide. Glycosphingolipids of the brain typically have a galactose attached to the ceramide, while non-neuronal tissue favors glucose addition (Baumann and Pham-Dinh, 2001).

#### Sphingolipid Synthesis

**Figure 5:**
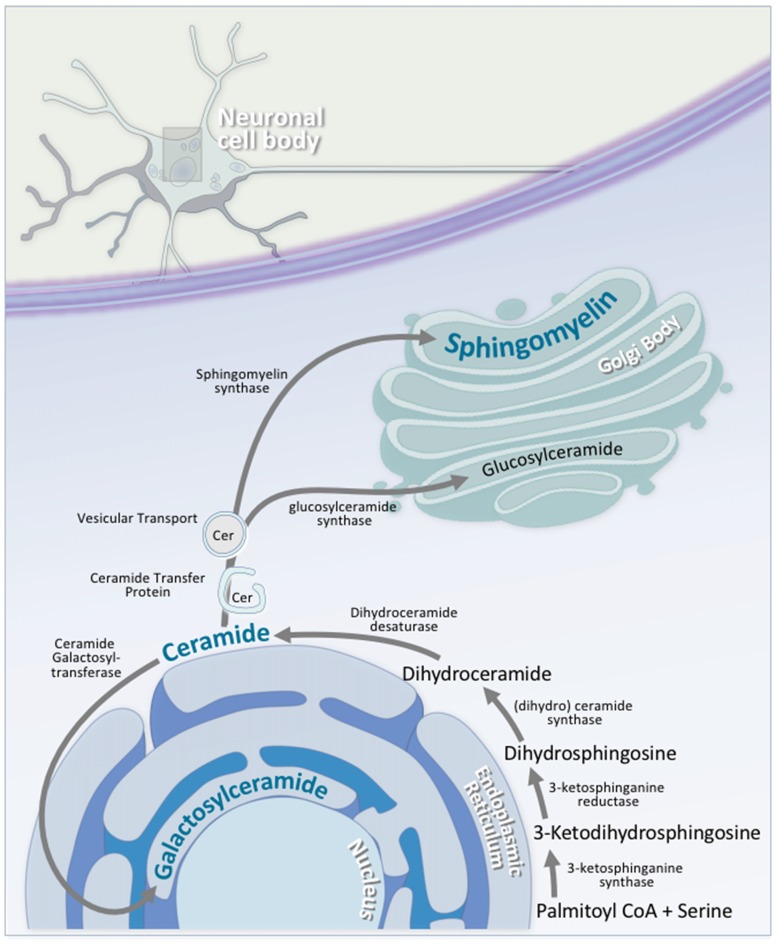
Neuronal sphingolipid synthesis takes place across multiple cellular compartments. Sphingolipid synthesis begins at the cytosolic leaflet of the ER. Via a series of reactions, palmitoyl CoA and serine are converted to ceramide. A portion of this ceramide is transported to the luminal leaflet of the ER, where ceramide galactosyltransferase (CGT) converts the ceramide to galactosylceramide; an essential neuronal sphingolipid. Another portion of this ceramide is transported to the Golgi complex, where it is converted to either glucosylceramide on the cytosolic side of the Golgi via glucosylceramide synthase, or to sphingomyelin on the luminal side by sphingomyelin synthase. Transport of ceramide from the ER to the Golgi complex is facilitated by either ceramide transfer protein (CERT) or vesicular transport.

Sphingolipid synthesis begins at the cytosolic leaflet of the ER, and progresses to several subcellular locations (Gault et al., 2010; Figure 5). At the ER, this process begins with the condensation of palmitoyl CoA and serine to 3-ketosphinganine. 3-ketosphinganine is then reduced to dihydrosphingosine by 3-ketosphinganine reductase. A family of (dihydro)ceramide synthases convert dihydrosphingosine to dihydroceramide. Ceramide synthase 1 is highly expressed in neurons in the brain, while ceramide synthase 2, 5, and 6 are expressed at lower levels (Becker et al., 2008; Laviad et al., 2008). Finally, dihydroceramide desaturase converts dihydroceramide to ceramide. At this stage, ceramide is either used by the cell, or transported elsewhere for further modification. A small portion of ceramide is transported to the luminal leaflet of the ER, where galactosylceramide is generated by ceramide galactosyltransferase (CGT). Galactosylceramide is particularly enriched in the CNS. It is highly expressed in Schwann cells and oligodendrocytes, and is also expressed, albeit at lower levels, in spinal, cerebellar, and brainstem neurons (Schaeren-Wiemers et al., 1995). The remaining ceramide is transported to the Golgi complex, where one of two enzymes catalyzes the synthesis of two complex sphingolipids. On the cytosolic side of the Golgi, glucosylceramide synthase (GCS) converts ceramide to glucosylceramide through the addition of a UDP-glucose group (Basu et al., 1968, 1973; Jeckel et al., 1992; Yamashita et al., 1999). On the luminal side of the Golgi, sphingomyelin synthase (SMS) converts ceramide to sphingomyelin through the addition of a phosphocholine group (Huitema et al., 2004; Figure 5). Two forms of sphingomyelin synthase exist; SMS1 and SMS2, and both are found on the trans-Golgi. SMS2, however, is also found on the plasma membrane, suggesting that it may play some role in sphingomyelin metabolism at the plasma membrane (Ternes et al., 2009; Yeang et al., 2011).

#### Sphingolipid Transport

The transport of ceramide from the cytosolic to luminal surface of the ER is currently not understood. It is hypothesized to occur through spontaneous intrabilayer transport or via protein-mediated transport (Hanada et al., 2003; Lopez-Montero et al., 2005; Hanada, 2010). Despite the suggestion of protein-mediated transport, no transporter proteins have been implicated to date. Transport from the luminal to cytosolic surface of the Golgi complex, however, has been shown to be dependent on non-vesicular FAPP2 transport protein (D’Angelo et al., 2013).

Transport from the ER to the Golgi complex occurs via two pathways (Figure 5). The first pathway involves the ceramide transfer protein (CERT), which transports ceramide in a non-vesicular, ATP-dependent fashion. It shows very high affinity for ceramide, and almost exclusively transports this ceramide for sphingomyelin synthesis (Hanada et al., 2003). Consequentially, it contains a number of highly regulated domains, which control this transport process (Levine and Munro, 1998; Loewen et al., 2003). The second pathway is less understood, and involves vesicular transport. Ceramide tracking studies have shown that vesicular transport machinery knockdown leads to defective transport of ceramide derivatives (Fukasawa et al., 1999), but direct evidence for vesicular trafficking is lacking.

## The Functions of Lipids in Neurons

### Lipids as Energy Substrates

The brain is an incredibly energy hungry organ. As such, it requires a near constant source of metabolites to maintain function. The general consensus is that this energy requirement is almost entirely satisfied by glucose metabolism. However, it has recently been shown that approximately 20% of the total energy requirement of the brain is met through the oxidation of fatty acids, and that this fatty acid oxidation may take place entirely in astrocytes (Ebert et al., 2003).

#### Pre-oxidation Steps

Fatty acid utilization for energy occurs through fatty acid oxidation, which takes place in the mitochondrial matrix. In order to be oxidized, fatty acids are converted to fatty acyl-CoA by acyl-CoA synthases—with the subtype of enzyme varying by fatty acid composition (Eaton et al., 1996). Once this reaction takes place, the substrates are transported across multiple mitochondrial membranes to the mitochondrial matrix. This process is undertaken by a set of proteins known as carnitine palmitoyltransferases (CPTs; McGarry and Brown, 1997). CPT1 on the outer mitochondrial membrane converts fatty acyl-CoAs into fatty acylcarnitines, which are transported into the intramembrane space through porins. CPT1 action is the rate-limiting step of fatty acid oxidation, and is regulated by malonyl-CoA. This is particularly important, because at this stage, fatty acyl-CoAs can either be directed to oxidation for fuel in times of energy requirement, or to the formation of structural glycerophospholipids in times of energy excess. Acylcarnatine transferases then transport the fatty acylcarnitines across the inner mitochondrial membrane in exchange for free carnitine. CPT2 then reforms the fatty acyl-CoA. At this stage, the fatty acyl-CoAs are ready to enter the β-oxidation pathway (McGarry et al., 1977, 1978; McGarry and Brown, 1997; Violante et al., 2010).

#### Mitochondrial β-Oxidation

Through a repeating sequence of four reactions that are catalyzed by acyl-CoA dehydrogenase, enoyl-CoA hydratase, hydroxyacyl-CoA dehydrogenase, and ketoacyl-CoA thiolase, β-oxidation produces a considerable amount of energy from a single substrate (Eaton et al., 1996). Each cycle of reactions produces one molecule of FADH_2_, one molecule of NADH, one molecule of acetyl CoA, and a fatty acyl derivative that is two carbons shorter than that in the previous step. This reaction cycle repeats until the entire carbon backbone has been broken down. In cases of an odd numbered carbon chain, propionyl-CoA is produced as the final product (Mazumder et al., 1963; Jenkins et al., 2015). The FADH_2_ and NADH incorporate their free electrons directly into the mitochondrial electron transport chain for ATP generation, and the acetyl CoA molecules enter the tricarboxylic acid cycle for further energy generation. This results in the generation of a significant amount of ATP (Lehninger, 1964; Balaban, 1990). Propionyl-CoA, through a further three step catabolic pathway, is converted to succinyl-CoA (Smith and Monty, 1959; Mazumder et al., 1961), which acts as a substrate for gluconeogenesis via oxaloacetate formation.

#### Peroxisomal β and α-Oxidation

Branched and very long-chain fatty acids are oxidized in the peroxisomes through the β-oxidation pathway (Lazarow and De Duve, 1976; Mannaerts and van Veldhoven, 1996). For long-chain fatty acids, the catabolism process occurs as in the mitochondria, with a few minor differences. Since peroxisomes do not have a tricarboxylic acid pathway, the acetyl-CoA/propionyl-CoA metabolites are transferred back to the mitochondria for degradation. Similarly, the peroxisomal β-oxidation pathway does not fully degrade fatty acids, only chain-shorten them. Non-saturated and branched fatty acids are metabolized via slightly different mechanisms. Due to the variation in substrate structure, a number of ancillary enzymatic complexes are required for catabolism. As a whole, the enzymes involved are multifunctional, with a wide range of substrate specificities (Hiltunen and Qin, 2000; Mannaerts et al., 2000; Poirier et al., 2006).

A small amount of fatty acid oxidation, termed α-oxidation, also occurs in the peroxisomes. α-oxidation removes a single carbon from fatty acids that are incapable of typical β-oxidation. For example, phytanic acid, which has a branched methyl group on the third carbon, is metabolized to pristanic acid through the α-oxidation pathway, allowing it to then be metabolized through the β-oxidation pathway (Singh et al., 1992, 1993; Pahan et al., 1993; Jansen and Wanders, 2006). α-oxidation is particularly important in the brain, as build-up of phytanic acid causes neurological damage, as is seen in Refsum’s disease (Jansen et al., 1997).

#### Regulating Fatty Acid Oxidation

Although the rate-limiting step of fatty acid oxidation occurs through CPT1, significant regulation also occurs as the result of the energy sensing capacity of the cell. The ratio of [NADH] to [NAD^+^], which serves as a measure of cellular energetic status, regulates the activity of hydroxyacyl-CoA dehydrogenase (Eaton et al., 1998). Low ATP levels activate AMPK, leading to the inhibition of lipogenic enzymes (Hopkins et al., 2003). This pathway is also regulated via the action of specific transcription factors. Peroxisome proliferator-activated receptor α (PPARα), which is triggered in low energy states, increases the expression of a number of catabolic enzymes, such as the CPT family, and acyl-CoA dehydrogenases (Leone et al., 1999). Low blood glucose triggers the activation of CREB, activating a number of essential lipid catabolic enzymes (Herzig et al., 2003).

#### Rationalizing Fatty Acid Oxidation

β-oxidation of palmitic acid produces seven FADH_2_, seven NADH and eight acetyl CoA molecules. These, in turn, produce 108 ATP molecules, giving a net gain of 106 ATP from a single molecule of palmitate (Reddy et al., 2014). By contrast, the metabolism of glucose yields at most, 36 ATP per glucose molecule (Hinkle et al., 1991). There is a staggering discrepancy between the amount of energy liberated from these molecules and the frequency of use of these pathways.

The brain is a very fragile organ, with small changes in environmental factors causing major disruptions. While accurate measurements of brain oxygen concentration are currently infeasible, the overall value can be described as low and nonuniform (Ndubuizu and LaManna, 2007). As anerobic glycolysis has low capacity for ATP generation, oxygen becomes the limiting factor. In this regard, the oxidation of 1 mol of palmitic acid requires 31 mol of oxygen, while oxidation of glucose requires 6 mol. Therefore, while palmitic acid may produce more ATP, it consumes significantly more oxygen to do so.

The consumption of oxygen also liberates a number of harmful products, to which the brain is particularly vulnerable. Reactive oxygen species, particularly in the form of superoxide, are generated in significant quantities from the β-oxidation pathway. Within the mitochondria, electron leakage at various steps in the electron transport chain produces superoxide radicals. Due to the large number of electrons being channeled through this pathway via NADH/FADH_2_, significant superoxide production occurs in this manner (Han et al., 2003; Turrens, 2003; Murphy, 2009; Perevoshchikova et al., 2013). Although most superoxide radical formation occurs at the mitochondria, peroxisomal β-oxidation also contributes. Free fatty acids have also been shown to be capable of binding to electron transport chain complexes, simultaneously decreasing the rate of oxidative ATP generation, while increasing superoxide generation by complexes I and III (Wojtczak and Schönfeld, 1993; Di Paola and Lorusso, 2006).

Therefore, while fatty acids are an ATP rich source of energy for the brain, the inadvertent reliance on glucose, along with oxygen-centric functionality of the brain requires that glucose must be used as the obligate substrate for energy production. In cases of fasting or extreme exertion, fatty acid utilization can increase, but sustained fatty acid oxidation in this manner only serves to damage the brain.

#### Ketone Bodies

During intense periods of fasting, fatty acid derivatives can be used in another, less harmful manner for energy. This process typically takes place in the liver, but has also been observed in brain astrocytes. In conditions of low glucose, fatty acid-derived acetyl-CoA is preferentially shuttled into the ketogenesis pathway to form three major ketone bodies; acetoacetate, D-3-β-hydroxybutyrate and propanone (Garber et al., 1974; Balasse and Neef, 1975; McPherson and McEneny, 2012). After synthesis, these ketone bodies enter the bloodstream, and are transported to peripheral targets, of which the major target is the brain (Owen et al., 1967). Upon reaching their target, they undergo ketolysis. Reversion of acetoacetate back to acetoacetyl-CoA (Serra et al., 1993) allows for the subsequent generation of acetyl-CoA, which then enters the tricarboxylic acid cycle to produce ATP (McPherson and McEneny, 2012).

The rationale behind ketogenesis is that it is primarily promoted by extrahepatic glucose levels, not necessarily the levels at the peripheral tissue (Robinson and Williamson, 1980). As such, the production of ketone bodies occurs at a site distant from the target. This is of particular importance to the brain, as acetyl-CoA formation, which is largely through β-oxidation, produces a host of damaging oxidative by-products. By limiting acetyl-CoA/ketone body synthesis to the liver, the brain decreases oxidative stress, while increasing available energy.

### Lipids as Cellular Structural Machinery

Perhaps the most essential role for lipids in the brain is as components of cellular structural machinery. This is particularly important in the brain due to the compartmentalization of the many signaling processes. While the major site of action of these lipids is the plasma membrane, they constitute all membranes found within the cell (van Meer et al., 2008). Lipid classes that take place in such functionality are the phospholipids, sterol lipids and sphingolipids. The common characteristic of these lipids is that they are amphipathic, allowing them self-organize in aqueous environments to form lipid bilayers. Each lipid class however, also has a unique role in membrane structure and function.

#### Phospholipids and Cellular Membranes

Phospholipids account for the majority of structural lipids in eukaryotic membranes. They form the major structural unit; the phospholipid bilayer. They are heavily implicated in the plasma membrane, along with the Golgi, ER, endosomes and mitochondrial membranes. Each of the subclasses play individual roles, with varying structural characteristics imparting functional variation.

Phosphatidylcholine is the most abundant of the phospholipids in cell membranes. Phosphatidylcholine has an almost perfect cylindrical molecular geometry. As a result, membranes composed of phosphatidylcholine do not feature any curvature (Thiam et al., 2013). While membranes consisting of phosphatidylcholine can be fluid or solid/gel-like, they are typically fluid at room temperature. By altering the ratios of phosphatidylcholine to other membrane phospholipids, the shape and permeability of the membrane can be altered. Modification of phosphatidylcholine to phosphatidic acid or lysophosphatidylcholine can also force the membrane into alternate geometries (van Meer et al., 2008). In the brain, the majority of choline used for neurotransmission is stored in the membrane as phosphatidylcholine (Blusztajn et al., 1987). As such, it serves as a vital reservoir for essential brain function.

Phosphatidylethanolamine is a minor component, and, for the most part, is found on the inner leaflet of the plasma membrane (Fadeel and Xue, 2009). Due to the relatively small head group, membranes with phosphatidylethanolamine assume a conical geometry, with significant outwards curvature (Thiam et al., 2013). Increases in phosphatidylethanolamine concentration also increase the fluidity of a membrane (Li et al., 2006). This is due to the nature of the fatty acyl chain, which is enriched in the PUFA arachidonic acid. In the brain, arachidonic acid is an essential precursor to a number of important neuromodulatory molecules, such as the prostaglandins and anandamides. The increased curvature and fluidity of the membrane introduced by phosphatidylethanolamine is hypothesized to facilitate vesicular budding and membrane fusion, two essential neuronal processes (Glaser and Gross, 1995; Lohner, 1996).

Phosphatidylserine is found largely on the inner leaflet of the plasma membrane (Fadeel and Xue, 2009). As a negatively charged phospholipid, phosphatidylserine is thought to act as an electrostatic mediator for a number of proteins (Maksymiw et al., 1987; McLaughlin and Aderem, 1995). Phosphatidylserine may also act as a buffer for essential bioactive fatty acids. Docosahexaenoic acid, which accounts for as much as 40% of all PUFAs in the brain, is essential for brain development and function, and is stored largely as phosphatidylserine (Guo et al., 2007; van Meer et al., 2008). It is therefore hypothesized that phosphatidylserine in membranes acts as a release/storage facility for docosahexaenoic acid.

Phosphatidylglycerol, in the context of eukaryotic membranes, does not play a major role. A small component of phosphatidylglycerol is observed in eukaryotic mitochondrial membranes (de Kroon et al., 1997; Morita and Terada, 2015). Cardiolipin, as a metabolite of phosphatidylglycerol, is a major constituent of mitochondrial membranes, accounting for as much as 15% of all lipids. In the mitochondria, cardiolipin maintains the membrane potential of the inner mitochondrial membrane, while also supporting proteins involved in mitochondrial respiration (Jiang et al., 2000). Phosphatidylinositol does not play a major role in membrane structure. It does, however, play major roles in membrane-bound signaling processes and vesicular activity, which will be discussed in the following section.

#### Sphingolipids and Cellular Membranes

The structural role of sphingolipids in membranes facilitates their role in signaling processes. The hydrophilic head groups contain a number of hydroxyl groups, which allow for extensive hydrogen bonding between individual head groups (Pascher, 1976; Boggs, 1987). This creates a flexible surface membrane that is largely impermeable. The fatty acyl groups that are associated with sphingolipids allow for thicker and more closely packed membranes. As a result, sphingolipids act as determinants of membrane fluidity and permeability (Pascher, 1976). A concentration gradient of sphingolipids is observed in cellular membranes. The ER has a low concentration, the Golgi has an intermediate concentration, and the plasma membrane and endosomes have a high concentration. This gradient is in place to align with cellular function. The ER has a low concentration since a more fluid membrane allows for easier protein insertion and folding, whereas a high sphingolipid concentration in the plasma membrane and endosomes creates thicker and less permeable barriers to outside molecules (van Meer et al., 2008).

Another structural component that sphingolipids take part in are lipid rafts. These lipid rafts are the result of the strong intermolecular forces between individual sphingolipid molecules, driving a phase separation of the sphingolipids from the phospholipid-rich outer membrane (Brown and London, 2000; Bacia et al., 2005). Present on membranes with high concentration of sphingolipids and cholesterol, lipid rafts act as major anchoring sites for proteins. Proteins that integrate with these rafts have been implicated in a host of processes, ranging from endocytic pathway sorting to antigen-responsive signaling (Posse de Chaves and Sipione, 2010).

#### Sterol Lipids and Cellular Membranes

Cholesterol plays a major role in determining cellular membrane flexibility and permeability. This is achieved through complex interactions of cholesterol molecules with the phospholipid bilayer. The structurally rigid planar ring structure—the sterol group, is the major facilitator of this (de Meyer and Smit, 2009). The polar nature of this group causes close interaction of the cholesterol molecules with phospholipids. This causes a condensation effect, whereby the lipid bilayer in these regions becomes tightly packed and ordered, creating a lipid ordered (l_o_) phase (Ege et al., 2006; Ali et al., 2007). In this phase, the membrane is still considered to be fluid, but the lipids within are in a much more ordered orientation. Such condensation also decreases membrane permeability in these regions (Bastiaanse et al., 1997). Interestingly, the association between phospholipids and cholesterol is dependent on phospholipid subtype. Phosphatidylcholine is the most highly associated, followed by phosphatidylserine and phosphatidylethanolamine. This is due to the nature of their sidechains, where cholesterol prefers to associate with saturated fatty acyl chains, to promote closer packing (Ohvo-Rekilä et al., 2002).

Depending on both the concentration of cholesterol, as well as the temperature of the membrane, cholesterol can have differing effects. At low concentrations cholesterol has a minor effect on membrane composition, and most phospholipid membranes are in a lipid disordered state. As cholesterol concentration increases, the membrane becomes more ordered, until crystallization begins to occur (Bach and Wachtel, 2003). At high temperatures, the tight packing of fatty acyl chains with cholesterol decreases the fluidity of the membrane, while at low temperatures, the presence of cholesterol hinders the tight packing that is required for highly ordered membranes (Khan et al., 2013). Thus, cholesterol acts as a buffer for temperature-dependent membrane fluidity, limiting the extremes typically observed in a cholesterol-free membrane. Despite these biophysical effects of cholesterol, the exact mechanism behind them is still unknown.

Cholesterol and sphingolipids also show close associations in the brain through lipid rafts. Along with the phase separation observed as the result of sphingolipid association, it is also understood to occur as the result of close associations between sphingolipids and cholesterol. A number of calorimetric and cholesterol partitioning experiments have shown that the affinity of cholesterol for sphingolipids is above that of phospholipids due to the amide linkage found in sphingolipids. Therefore, such close associations drive further phase separation between the sphingolipids and phospholipids, promoting the formation of these raft structures. Furthermore, the liquid ordered state, as facilitated by cholesterol, is hypothesized to be the phase required for lipid raft formation (Silvius, 2003).

### Lipids as Bioactive Molecules

As bioactive molecules, lipids take part in a wide range of cellular signaling processes. Here, signaling processes will only be reviewed in the context of the CNS. Fatty acids and their derivatives have been well characterized as drivers of intracellular signaling processes (Graber et al., 1994). One class that show particularly well-defined roles are the PUFAs. As previously mentioned, the brain is enriched in two major PUFAs; arachidonic acid and docosahexaenoic acid. Consequentially, PUFAs have been implicated in neuronal signaling processes controling neurogenesis, brain vesicular activity, central glucose homeostasis, mood and cognition (Bazinet and Layé, 2014).

Unmodified PUFAs primarily act upon fatty acid-activated receptors. The most well studied family of receptors are the PPARs. In the brain, PPARδ and PPARβ are involved in the regulation of fatty acid metabolism and inflammatory responses (Tyagi et al., 2011). PUFAs also downregulate SREBP1 activity, which is involved in *de novo* lipogenesis (Infantino et al., 2007). This effect is further enhanced by the action of PUFAs on liver X and retinoid X receptors (LXRs and RXRs), where PUFA binding inhibits SREBP1 activation via LXR/RXR (Yoshikawa et al., 2002).

PUFAs are also involved in more distinct signaling pathways. Endocannabinoids are fatty acid derivatives, with the major forms in the brain being the arachidonic acid derivatives anandamide, and 2-arachidonoylglycerol. These bind to cannabinoid receptor type 1 and 2 on both neurons and glia (Matsuda et al., 1990; Tsou et al., 1998; Howlett and Mukhopadhyay, 2000). Acting as retrograde messengers at type 1 receptors, they supress neurotransmitter release (Kim and Thayer, 2000). At excitatory and inhibitory synapses this mediates short-term synaptic plasticity and long term depression (Gerdeman et al., 2002; Chevaleyre and Castillo, 2003; Kano et al., 2009). While this occurs largely on neurons, endocannabinoids have been shown to mediate these effects through glial cell receptors (Hong et al., 2003).

PUFAs also play a major role in inflammatory signaling pathways. Interestingly, the structure of the PUFA can significantly alter inflammatory response, where omega-3 fatty acids have an anti-inflammatory effect in the brain (Calder, 2010), and omega-6 fatty acids have a pro-inflammatory effect (Patterson et al., 2012). Consequentially, expression of docosahexaenoic acid and its intermediates have been shown to have a potent anti-inflammatory effect by lowering levels of pro-inflammatory cytokines in the brain following LPS administration (Delpech et al., 2015). Studies have also shown that diets rich in docosahexaenoic acid lower the risk of neuroinflammatory diseases (Minogue et al., 2007). Arachidonic acid intermediates, however, are potent neuroinflammatory enhancers. Major metabolites of arachidonic acid are the prostaglandins, which have been heavily implicated in inflammatory responses throughout the body. Their expression is particularly high under pathogenic neuroinflammatory conditions, suggesting a critical role in brain pro-inflammatory responses (Ricciotti and FitzGerald, 2011; Lima et al., 2012).

Phosphorylated forms of phosphatidylinositol activate phospholipase C, creating inositol triphosphate (IP_3_) and diacylglycerol (Berridge and Irvine, 1984; Vanhaesebroeck et al., 2001). IP_3_ is transported rapidly to the cytosol where it promotes calcium release (Berridge, 2009). In this way, phosphatidylinositol signaling in the brain has been linked to inter-neuronal communication through vesicular-mediated action of muscarinic and serotonergic receptors. Diacylglycerol can either be phosphorylated to give the phospholipid precursor phosphatidic acid (Rodriguez de Turco et al., 2001), or hydrolyzed to arachidonic acid precursors (Bell et al., 1979). In this way, diacylglycerol can give rise to a host of signaling processes, through two diverging pathways.

Sphingolipids also play a major signaling role in the brain. The brain contains a high concentration of gangliosides, which are synthesized through the addition of sialic acid to glycosphingolipid monomers (Yu et al., 2011). In neuronal membranes, gangliosides make up as much as 12% of total lipid content (Posse de Chaves and Sipione, 2010). Throughout development, the composition of brain gangliosides switches from predominantly simple gangliosides (GM3) to complex gangliosides (GM1a). Such changes in the expression patterns of gangliosides suggest a role in brain development (Yu et al., 1988; Ngamukote et al., 2007). Taken together, gangliosides have been shown to have major roles in membrane protein modulation, cell-cell adhesion, axonal growth, synaptic transmission, neural development and differentiation and receptor regulation (Yu et al., 2011).

In many cases, a combination of lipids facilitates signaling events. This is particularly the case for lipid rafts. The close association of phospholipids, cholesterol and sphingolipids leads to the formation of lipid rafts (Simons and Sampaio, 2011). Lipid rafts serve as major organizing centers for proteins and signaling molecules, acting as essential cellular signaling components (Allen et al., 2007). In the brain, lipid rafts have been implicated in ionotropic receptor localization, binding and trafficking, neurotransmitter transport, cytoskeletal rearrangement through tubulin and actin remodeling, exocytosis, organization of G-protein coupled receptor machinery assembly for downstream signaling, cell surface receptor clustering, metabolism, neuronal growth and development, and redox signaling (Allen et al., 2007; Benarroch, 2007; Jin et al., 2011). Thus, lipid rafts are the central organizing space for all major classes of neuronal processes.

In summary, neuronal lipid signaling occurs through a range of processes, some driven by individual lipid classes, while others require more complex associations. The result is an incredibly intricate system that has multiple layers of redundancy, ensuring tightly controlled processes.

## Neuronal Lipid Metabolism in Amyotrophic Lateral Sclerosis

### Amyotrophic Lateral Sclerosis

ALS is a progressive neurodegenerative disorder that is characterized by the selective degeneration of upper motor neurons in the motor cortex and lower motor neurons in the brainstem and spinal cord. The progressive degeneration of these motor neurons leads to paralysis, and eventual death within 2–5 years from diagnosis (Kiernan et al., 2011). Despite the breadth of research on ALS, its etiology is still not well understood. A growing number of *in vitro* and *in vivo* studies have begun to investigate metabolism as a means of explaining the neuropathology observed in ALS. While a number of metabolic hallmarks have been observed in ALS patients (Reyes et al., 1984; Desport et al., 2005; Dupuis et al., 2008; Pradat et al., 2010; Jésus et al., 2018), interesting alterations in lipid handling mechanisms have also been noted to occur (Dupuis et al., 2008; Dorst et al., 2011).

A major site of interest for lipid studies in ALS is skeletal muscle. Many studies have suggested that skeletal muscle is a major source of dysregulated lipid metabolism. Indeed, a defined switch from glucose-based to lipid-based metabolism is an early pathological event in ALS muscle (Palamiuc et al., 2015). Furthermore, significant alterations in glycosphingolipid metabolism in the muscle of ALS mice impacts muscle innervation and motor recovery (Henriques et al., 2015b, 2017). Thus, dysregulation in lipid metabolism in skeletal muscle have been linked to pathological outcomes.

### CNS-Specific Alterations in Lipid Metabolism in ALS

Having reviewed the multiple functions of lipids individually, we will now frame the dysfunctions caused by abnormal lipid metabolism in ALS in this way.

#### Dysfunctions in Lipids as an Energy Substrate

A growing focal point in ALS research is the role of lipids as an energy substrate. Given consistent observations of altered lipid metabolism in skeletal muscle, research has begun to consider neuronal lipid energy use in ALS. Such research, however, is still in its infancy. Perhaps the most compelling evidence towards a pathological role for lipid metabolism in ALS neurons is through CNS-specific oxidative stress, in which a range of lipid-derived oxidative pathway intermediates have been observed at heightened levels in the CNS (Tohgi et al., 1999; Bogdanov et al., 2000; Simpson et al., 2004; Mitsumoto et al., 2008). With the discovery of the superoxide dismutase-1 (SOD1) mutant in ALS, researchers were quick to pin the cause of oxidative stress on this mutation (Rosen et al., 1993; Wiedau-Pazos et al., 1996; Andrus et al., 1998). Further studies have determined that while the SOD1 mutation may contribute to oxidative stress, it is not the major cause. This is supported by the presence of oxidative stress in non-SOD1 ALS (Duan et al., 2010; Braun et al., 2011; Iguchi et al., 2012; Kiskinis et al., 2014; Carri et al., 2015; Hirano et al., 2015; Zhan et al., 2015).

**Figure 6:**
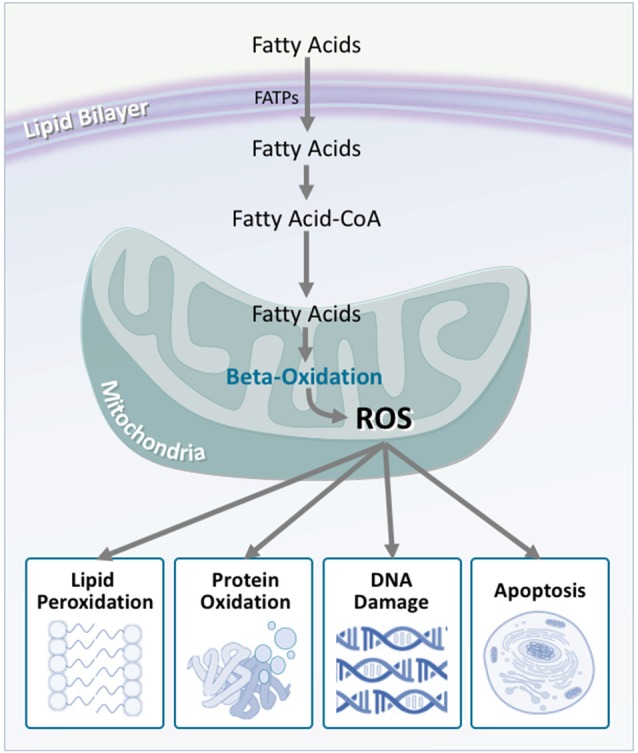
Fatty acid oxidation is a major contributor to reactive oxygen species production, which is increased in amyotrophic lateral sclerosis (ALS). Although fatty acids are not the obligate substrate for energy production in the cell, β-oxidation of fatty acids generates a substantial amount of reactive oxygen species as a by-product. In turn, these promote a number of harmful oxidative effects including lipid peroxidation, protein oxidation, DNA damage, and apoptosis. As neurons are not effectively equipped to deal with oxidative stress, these harmful effects are multiplied, contributing to neurodegeneration.

In light of this, researchers have considered energetic substrate metabolism as a source of oxidative stress. In the brain, neuronal metabolism is largely aerobic, and involves glucose/lactate, while glial cell metabolism is anaerobic (Itoh et al., 2003; Herrero-Mendez et al., 2009). During normal neuronal activity, oxidative stress is kept relatively low (Almeida et al., 2004). In ALS, however, an increased demand for energy is placed on the motor neurons. Despite this, brain and spinal cord glucose use (Hatazawa et al., 1988; Browne et al., 2006), as well as the concentration of tricarboxylic acid cycle intermediates are significantly decreased (Niessen et al., 2007). Similarly, reduced lactate transport and metabolism (Lee et al., 2012), damaged neuronal mitochondria (Magrané et al., 2014), and mitochondrial electron transport chain dysfunction (Crugnola et al., 2010; Shi et al., 2010; Smith et al., 2017) highlight that in ALS, the brain has a significantly decreased ability to metabolize glucose for fuel. It is therefore hypothesized that alternate substrates are metabolized to meet the energy requirements of the brain. Indeed, in mouse models of ALS, lipid catabolism and clearance to peripheral tissues is significantly increased (Fergani et al., 2007; Dodge et al., 2013). Similarly, elevated levels of ketone bodies have been observed in ALS patient cerebrospinal fluid (Blasco et al., 2010; Kumar et al., 2010). With the suggestion of increased peripheral lipid availability, it is plausible that metabolic utilization of these lipids would increase. Consequentially, studies of mouse models of ALS, as well as in ALS patients, show that markers of oxidative stress and lipid peroxidation are significantly elevated in brain and spinal cord tissue, via lipid-centric pathways (Simpson et al., 2004; Miana-Mena et al., 2011). Hence, an increased focus on lipid metabolites as a fuel source would inevitably lead to increased oxidative stress, which would have number of deleterious outcomes (Figure 6).

In ALS patient muscle, peroxisome proliferator-activated receptor gamma coactivator 1-α (PGC-1α), a master regulator of normal mitochondrial function and biogenesis, is downregulated, leading to modifications in fatty acid signaling, and increased β-oxidation (Barroso et al., 2011; Thau et al., 2012). In mouse models of ALS, downregulation of PGC-1α has been shown to hasten disease progression (Eschbach et al., 2013), while upregulation of PGC-1α has been shown to maintain healthy levels of mitochondrial biogenesis in muscle, as well as improve muscle function (Da Cruz et al., 2012). Survival, however, is not extended. Together, these findings highlight the essential link between fatty acid oxidation and disease, while suggesting that muscle may not be the primary target. Application of these findings to a neuronal model, therefore, would be expected to have more drastic effects, given the poorer oxidative defense capabilities of the CNS. Interestingly, when PGC-1α is upregulated in the CNS, mitochondrial function is not only improved centrally, but motor function and survival are also drastically improved (Zhao et al., 2011). Therefore, a strong case can be made for the role of PGC-1α in maintaining CNS-driven fatty acid metabolism. In a similar fashion, the stearoyl-CoA desaturase 1 (SCD-1) gene has been implicated in ALS. SCD-1 is a key enzyme in fatty acid metabolism regulation, and directly alters the levels of β-oxidation that occur in the mitochondria (Ntambi, 1999; Ntambi et al., 2002). In mouse models of ALS, as well as ALS patient muscle samples, SCD-1 has been shown to be downregulated (Pradat et al., 2012; Hussain et al., 2013). While downregulation of SCD-1 may explain increased expression of β-oxidation enzymes, increased energy expenditure, and reduced fat storage in ALS mice (Dupuis et al., 2004), studies have yet to consider the role of SCD-1 in the CNS.

#### Dysfunction in Lipids as Cellular Structural Machinery

In mouse models of ALS, it has been shown that membrane fluidity in the brain and spinal cord decreases significantly over the course of disease (Miana-Mena et al., 2011). There are a number of potential hypotheses for why this may occur. The first involves central PUFA concentrations. The brain contains a very high concentration of PUFAs, which are stored as phosphatidylethanolamine (arachidonic acid) or phosphatidylserine (docosahexaenoic acid) in neuronal membranes (Bazinet and Layé, 2014). Due to the highly unsaturated nature of these fatty acids, neuronal membranes rich in phosphatidylethanolamine and phosphatidylserine are significantly less fluid. In ALS, docosahexaenoic acid levels are significantly increased in the brain, which may result in more rigid membranes (Ilieva et al., 2007). Another theory that supports these findings involves lipid peroxidation. PUFAs are particularly sensitive substrates in lipid peroxidation reactions. Due to the highly oxidative environment of the brain in ALS, lipid peroxidation occurs at a higher rate. This is supported somewhat by the observation that high levels of lipid peroxidation intermediates exist in ALS patient spinal cord (Shibata et al., 2001). In the context of lipid membranes, peroxidation makes membranes less fluid through modification of the polyunsaturated/saturated lipid ratio, as well as promoting lipid-lipid and lipid-protein cross-linking (Rice-Evans and Burdon, 1993; Chen and Yu, 1994; Miana-Mena et al., 2011). Since a large majority of signaling lipids and proteins are found within membranes, it is possible that decreases in fluidity will decrease their mobility, impairing their function, and leading to pathological outcomes through interruptions to signaling pathways. Due to the limited number of studies in this area, further research is needed to determine the role of lipids in cellular structure and integrity in ALS.

#### Dysfunction in Lipids as Bioactive Molecules

Due to the diverse nature of lipid signaling in the brain, the potential for multifactorial pathways for lipid dysfunction is great. PUFAs are known to bind to a number of essential metabolic transcription factors, such as LXR, which regulate lipid levels in the CNS. Specifically, LXRs modulate cholesterol levels, acting as endogenous cholesterol sensors. In ALS, disruptions to LXR signaling have been implicated in dysfunctional signaling cascades, leading to motor neuron and glial cell damage in SOD1 mice. LXR knockout mice show neuroinflammatory responses leading to motor neuron loss, and neuromuscular junction defects (Mouzat et al., 2017). A number of PUFAs also act as pro- or anti-inflammatory signaling molecules. For example, the PUFAs eicosapentaenoic acid and arachidonic acid are oxidized to form prostaglandins or leukotrienes—essential central inflammatory molecules. In ALS patients, elevated levels of prostaglandin E2 are observed in serum and cerebrospinal fluid (Iłzecka, 2003). Furthermore, pharmacological inhibition of the prostaglandin E2 receptor (Liang et al., 2008) along with downregulation of pathway intermediates (Pompl et al., 2003) has also shown therapeutic benefits in SOD1 mice, suggesting that lipid-derived prostaglandin synthesis causes downstream pathophysiological outcomes.

In a broader sense, disruption in signaling can arise from more than fatty acid-centric pathways. Concurrent with lipid peroxidation affecting membrane fluidity, peroxidation also affects the composition of lipid rafts, and excitotoxic signaling pathways (Zhai et al., 2009). Lipid rafts act as major structures for protein binding and signaling in the CNS, suggesting that significant variation in raft composition may affect signaling processes through alterations in protein association. Indeed, proteomic studies on lipid raft composition in the spinal cord of SOD1 mice show a total of 67 differentially expressed proteins, with major roles in vesicular transport, neurotransmitter synthesis and release, cytoskeletal organization and metabolism (Zhai et al., 2009). In terms of excitotoxic outcomes, lipid peroxidation produces a number of damaging by-products, such as 4-hydroxynonenal. In ALS patients, higher levels of these molecules in the spinal cord has been linked with modification of the astrocytic glutamate transporter EAAT2 (Pedersen et al., 1998). Given that astrocytic EAATs play a key role in protecting against microglial glutamate-induced neuronal death, it is possible that reduced expression of EAAT2 and glutamate excitotoxicity in ALS (Rothstein et al., 1995; Bristol and Rothstein, 1996; Lin et al., 1998) may be strongly influenced by lipid activity. Interestingly, EAAT2 is a protein that is associated almost entirely with lipid rafts (Butchbach et al., 2004). Therefore, it stands to reason that changes in lipid composition, as observed in ALS, will significantly affect EAAT2 activity.

A curious finding amongst mouse models of ALS, as well as ALS patients, is an increase in sphingolipids in the central nervous system, due to oxidative stress. The initial assumption was that aberrant sphingolipid metabolism was causing a pathological upregulation of sphingolipid metabolites, leading to neurodegenerative outcomes (Cutler et al., 2002). More recently, it has been shown that glycosphingolipid metabolites are significantly increased in skeletal muscle, but decreased in the CNS (Dodge et al., 2015; Henriques et al., 2015b). Since pathological outcomes are observed in both tissue types, it is possible that glycosphingolipids exert their effects in a dose dependent manner, where chronically high or low levels affect signaling fidelity, leading to pathology (Dodge et al., 2015).

### Targeting Lipid Metabolism for ALS Treatment

In light of the proposed mechanisms for dysregulation of lipid pathways in ALS, treatments targeting these pathways have generated significant interest. High levels of circulating lipids and higher body mass index positively correlate with better prognosis and longer survival in ALS (Dupuis et al., 2008; Paganoni et al., 2011; Henriques et al., 2015a). As such a number of dietary interventions have been trialed for ALS treatment. High fat diets exert a modest decrease in disease progression in a mouse model of ALS (Dupuis et al., 2004; Mattson et al., 2007), while ketogenic diets improve motor function and mitochondrial activity without increasing survival (Zhao et al., 2006, 2012). Despite a large body of data to suggest that the adoption of high-calorie or high-protein diets may be of some benefit for ALS patients (Silva et al., 2010; Dorst et al., 2013; Wills et al., 2014; Ngo et al., 2017), large clinical trials that validate their effectiveness are lacking.

While druggable targets for lipid metabolism are plentiful, therapeutics to target these pathways in ALS remain largely untested. One promising therapeutic intervention so far relates to the modulation of the balance between fatty acid and glucose oxidation. It has recently been shown that SOD1 mice exhibit a preferential switch towards fatty acid oxidation, and that a reversal of this switch to promote glucose oxidation through treatment with dichloroacetate leads to significant improvements in motor function (Palamiuc et al., 2015). While Palamiuc et al. (2015) did not determine whether these improvements in motor function were due to neuroprotective effects on motor neurons, dichloroacetate has been shown to improve mitochondrial redox status in astrocytes, thereby preventing astrocyte-mediated toxicity, and rescuing motor neurons from death and improving survival in SOD1 mice (Miquel et al., 2012). Whether dichloroacetate improves redox status in motor neurons in ALS remains to be investigated.

Another compound that has shown promise is conduritol B epoxide, a potent β-Glucocerebrosidase that modulates sphingolipid metabolism. By increasing the levels of glucosylceramide in SOD1 mice, conduritol B epoxide not only attenuates the dysregulation of genes that are involved in pathogenic pathways, it also preserves neuromuscular junction function and rescues motor neurons from death in a mouse model of ALS (Henriques et al., 2017). In this regard, inhibition of glucosylceramide synthesis has been shown to hasten disease progression in SOD1 mice (Dodge et al., 2015). Thus, neuronal and muscular glycosphingolipids serve as an exciting target for further research and therapeutic development for ALS.

## Conclusion

**Figure 7:**
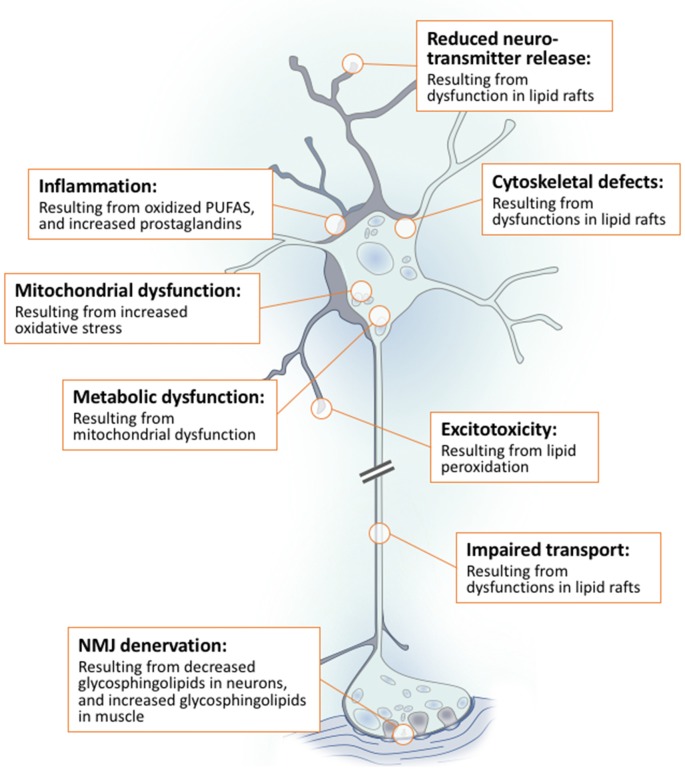
Dysregulated lipid metabolism exerts a multifaceted effect on neurons in ALS. Dysregulation of neuronal lipid metabolism in ALS impacts energy use, structural integrity and signaling processes. Increased use of lipid as an energy substrate leads to increased oxidative stress. This exacerbates inflammation, mitochondrial dysfunction, metabolic dysfunction and excitotoxicity. Altered lipid metabolism also disrupts intracellular lipids leading to cytoskeletal defects and the denervation of neuromuscular junctions. Finally, changes in lipid metabolism impacts the composition of lipid rafts. This disrupts signaling processes that are crucial in regulating neurotransmitter synthesis and release, cytoskeletal integrity and intracellular transport.

The neuronal metabolism of lipids is a system of great depth, with functional outcomes ranging from energy substrate availability through to nuanced signaling pathways. Despite this, it remains an area of many unknowns. In ALS, neuronal lipid metabolism is dysregulated in a number of ways, affecting energy use, structural integrity, and signaling processes. In terms of energy use, neurons metabolize a greater proportion of lipid substrates, increasing oxidative stress. This leads to inflammation, mitochondrial dysfunction, metabolic dysfunction and excitotoxicity. At a structural level, altered lipid metabolism disrupts intracellular lipid levels, leading to cytoskeletal defects, and neuromuscular junction denervation. From a signaling perspective, altered lipid metabolism affects the composition of lipid rafts, disrupting important signaling processes, leading to defects in neurotransmitter synthesis and release, cytoskeletal defects, and impaired intracellular transport (Figure 7).

While recent research into the role of glycosphingolipid metabolism in ALS has opened avenues for the development of potential novel therapeutics, more studies are needed to understand the functional consequences of alterations in lipid metabolism in ALS as a whole. This, in turn, will ultimately lead to more promising treatment opportunities, the beginnings of which are already proving to be fruitful.

## Author Contributions

TJT conducted the literature search and wrote the manuscript. FJS produced all artwork. FJS, EJW and STN critically reviewed the manuscript.

## Conflict of Interest Statement

The authors declare that the research was conducted in the absence of any commercial or financial relationships that could be construed as a potential conflict of interest.
